# Sphenopalatine-sphenopalatine anastomosis: a unique cause of intractable epistaxis, safely treated with microcatheter embolization: a case report

**DOI:** 10.1186/1752-1947-1-125

**Published:** 2007-10-31

**Authors:** Tawakir Kamani, Simon Shaw, Ahmed Ali, George Manjaly, Martin Jeffree

**Affiliations:** 1 Royal Albert and Edward Infirmary, Wigan, UK; 2 Royal Bolton Hospital, Bolton, UK; 3 Eastbourne District General Hospital, Eastbourne, East Sussex, UK; 4 Hurstwood Park Neurological Centre, Haywards Heath, UK

## Abstract

Epistaxis is the most common emergency presenting to the ENT surgeon. Here we present a case of epistaxis arising from the sphenopalatine artery in a patient who had previously had the ipsilateral external carotid artery ligated due to previous epistaxis. On investigation the epistaxis was determined to arise from an anastamosis with the contralateral sphenopalatine artery. The anatomy was demonstrated with angiography and the epistaxis treated using microcatheter embolization. Anatomical variation can be a cause for failure of ligation as a permanent treatment for epistaxis. Embolization is used less frequently for epistaxis control due to concerns about the risks involved, but it can be a valuable treatment option in intractable epistaxis following a failure of arterial ligation.

## Introduction

The commonest emergency presenting to the ENT surgeon is epistaxis (Small and Maran 1984) [1]. Only 10% of those who experience epistaxis seek medical attention and only 1% of these require surgical intervention (Ram et al, 2000) [2]. Initial management of posterior epistaxis is conservative with nasal packing and bed rest. Arterial ligation of any of the internal maxillary artery (IMA), anterior ethmoid artery (AEA), external carotid artery (ECA), or more recently endoscopic ligation of the sphenoplatine artery (SPA), are last resorts. Endovascular embolization of these feeder vessels has lost popularity due to advances in nasal endoscopy. Embolization is also associated with complications such as neurological injury, which do not occur with endoscopic SPA ligation.

However, angiography with endovascular embolization is a valuable tool for diagnosis of rare vascular anatomical variations and their subsequent treatment when ligation has failed. This has previously been demonstrated in a rare case on uncontrolled epistaxis secondary to vertebro-carotid anastomosis despite multiple arterial ligations [3]. We describe our experience of angiography and endovascular embolization in the treatment of a patient with a severe delayed recurrent posterior epistaxis after undergoing ipsilateral arterial ligation.

## Case report

A 70 year old hypertensive Caucasian female patient presented to the Emergency Unit with left sided epistaxis. She had a past medical history of left-sided epistaxis 4 years ago for which she initially underwent left sphenopalatine (SPA) endoscopic ligation. During this episode epistaxis recurred so she underwent left external carotid artery and left anterior ethmoid artery ligation. She was subsequently completely free of epistaxis for 4 years. Two months prior to the episode we present she suffered a further bleed, which was successfully treated with anterior nasal packing.

**Figure 1:**
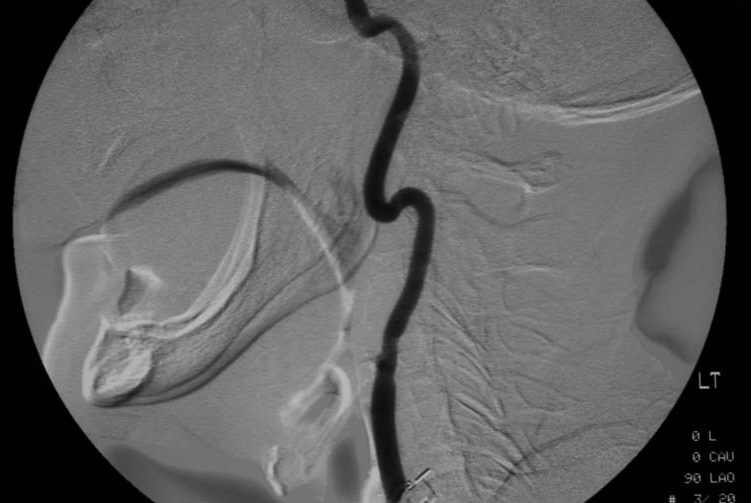
Left common carotid artery angiogram demonstrating external carotid ligation.

**Figure 2:**
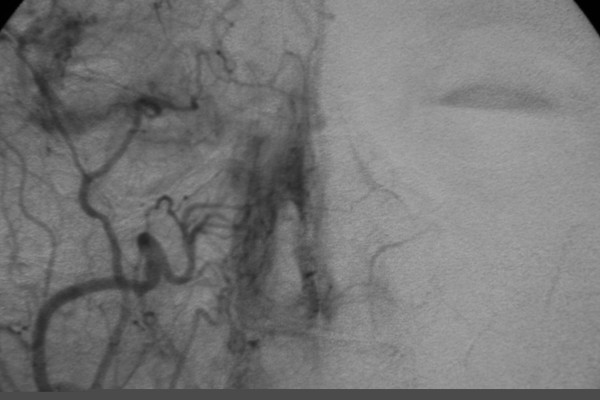
Selective right external carotid artery angiogram demonstrating collateralization with the left side.

During this presentation she was haemodynamically stable, with a blood pressure of 160/80 mmHg, and had normal coagulation screen, biochemistry and full blood count. During endoscopy, a bleeding point in the left SPA region was identified and bismuth iodoform paraffin paste (BIPP) nasal packing applied. She continued to bleed intermittently. Angiography was planned prior to further surgical intervention due to her past history of left SPA ligation. This confirmed complete occlusion of left ECA (figure 1). The right ECA demonstrated a few small nasal branches of the right SPA crossing to the left, with no evidence of a bleeding point initially (Figure 2). A literature search revealed that sphenopalatine-sphenopalatine anastomosis has not previously been reported.

**Figure 3:**
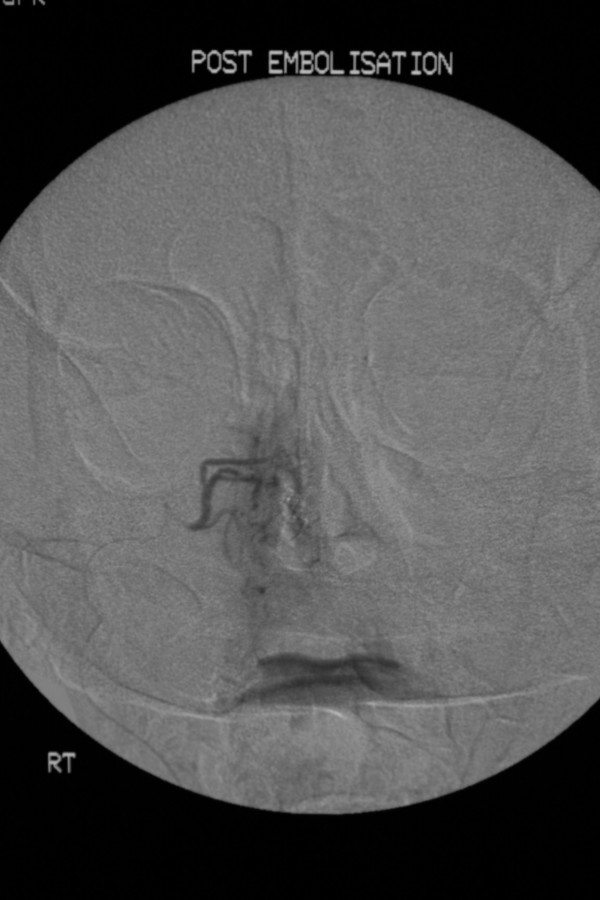
Post particle embolisation of the right sphenopalantine Artery demonstrating absence of pooling of contrast in the left nasal passage.

A microcatheter was advanced distally in the right SPA and angiography showed pooling of a small amount of contrast in the upper part of the left nostril. Embospheres in the 80–120 μ size range and PVA particles in the 355–500 μ range were injected into the right SPA. Subsequent angiography revealed a reduction in the number of the nasal branches and no further pooling (Figure 3). The nose was packed anteriorly at the end of the procedure. Packing was removed 24 hours later and the patient has had no further bleeding, and was hemorrhage free at follow up.

## Discussion

Traditionally, posterior epistaxis has been managed with a sequence of nasal packing, SMR, septoplasty, anterior ethmoid ligation, sphenopalatine artery ligation, transantral maxillary artery ligation, selective maxillary artery embolization and external carotid artery ligation. However, a single intervention is yet to provide definitive treatment.

The failure rates for ligation of the SPA (0–16%) [2] and the internal maxillary artery (5–15%) [4,5] are similar. These may have been attributed to the anterior ethmoid arteries not being ligated. In a previous study, it was noted that improved control of epistaxis occurred when anterior ethmoid artery occlusion was performed with internal maxillary ligation [6]. Failure has also been previously attributed to the variable branching of both IMA and SPA, making it difficult to find the correct vessel. However, it has also been shown that 13% of failures were due to incorrect placement of clips [4].

Embolization requires the expertise of an experienced interventional radiologist and has been associated with serious neurological complications including hemiplegia, ophthalmoplegia and facial paralysis at rates of 0–8% [7,8]. However, it offers excellent localization of the offending vessel and in our patient, demonstrated a unique anatomical anastomosis and allowed it to be successfully managed. Ipselateral external carotid artery ligation has the potential disadvantage of removing a route for future angiography and subsequent embolization. Fortuitously in this case, the communication between the right and left sphenopalatine arteries allowed successful diagnostic angiography and a route for embolization of the ipsilateral sphenopalatine artery via the contralateral external carotid artery.

## Conclusion

Due to complication severity and frequency, embolization in the control of epistaxis has been unpopular. However, in cases such as ours, when one is presented with a patient with recurrent and persistent epistaxis despite multiple ipsilateral ligations, angiography can delineate the vascularity of the nose and identify the bleeding vessel. Patients with such persistent bleeding may have a rare variation in vascular anatomy, as was seen in our patient. With a microcatheter, the offending vessel can be embolized.

## Competing interests

The author(s) declare that they have no competing interests.

## Authors' contributions

Jointly written by Kamani, T and Shaw, S. Revisions suggested by Ali, A. Manjaly G; and Jeffree MJ supervising authors.

## Consent

Informed written consent was received for publication of this manuscript.
